# Fatal tick-borne encephalitis virus infection in Dalmatian puppy-dogs after putative vector independent transmission

**DOI:** 10.1080/01652176.2024.2338385

**Published:** 2024-04-10

**Authors:** Kara L. D. Dawson, Giuliana Rosato, Simone Egloff, Carole Burgener, Anna Oevermann, Paula Grest, Monika Hilbe, Torsten Seuberlich

**Affiliations:** a Division of Neurological Sciences, Vetsuisse Faculty, University of Bern, Bern, Switzerland; b Institute of Veterinary Pathology, Vetsuisse Faculty, University of Zurich, Zurich, Switzerland; c Clinic of Reproductive Medicine, Vetsuisse Faculty, University of Zurich, Zurich, Switzerland

**Keywords:** Tick-borne encephalitis virus, puppies, metatranscriptomics, high-throughput sequencing

## Abstract

In a retrospective metatranscriptomics study, we identified tick-borne encephalitis virus (TBEV) to be the causative agent for a fatal non-suppurative meningoencephalitis in a three-week-old Dalmatian puppy in Switzerland. Further investigations showed that the two other littermates with similar signs and pathological lesions were also positive for TBEV. By using an unbiased approach of combining high-throughput sequencing (HTS) and bioinformatics we were able to solve the etiology and discover an unusual case of TBEV in three young puppies. Based on our findings, we suggest that a vector-independent transmission of TBEV occurred and that most likely an intrauterine infection led to the severe and fulminant disease of the entire litter. We were able to demonstrate the presence of TBEV RNA by *in situ* hybridization (ISH) in the brain of all three puppies. Furthermore, we were able to detect TBEV by RT-qPCR in total RNA extracted from formalin-fixed and paraffin embedded (FFPE) blocks containing multiple peripheral organs. Overall, our findings shed light on alternative vector-independent transmission routes of TBEV infections in dogs and encourage veterinary practitioners to consider TBEV as an important differential diagnosis in neurological cases in dogs.

## 1. Introduction

Tick-borne encephalitis virus (TBEV) is a single-stranded enveloped (+) RNA virus of the family *Flaviviridae*, genus *Flavivirus* (Simmonds et al. 2017). TBEV is an emerging zoonotic arbovirus and can cause severe neurological diseases in both humans and animals (Ruzek et al. 2019). The main transmission route is through bites by ticks of the genus *Ixodes* and less frequently through an alimentary route after the consumption of unpasteurized milk from infected goats, cows or sheep (Chitimia-Dobler et al. 2021). A significant increase of human TBEV infections in Europe has been observed over the last few decades, giving rise of attention towards disease incidences and its geographical spread (Schuler et al. 2014; Deviatkin et al. 2020; Kunze et al. 2021).

Dogs are frequently infected with TBEV without developing clinical signs; however, also in dogs an increase of fulminant TBEV cases has been observed (Leschnik et al. 2002; Pfeffer and Dobler 2011). It is mostly unknown why TBEV infections rarely lead to clinical manifestations with fatal outcomes in dogs. Several factors play a role in determining the clinical course of TBEV in dogs, including age, breed, immune status, and general health as well as the TBEV strain and the infectious dose (Salat et al. 2021).

In a retrospective metatranscriptomics study of unveiling the viral etiology of canine non-suppurative encephalitis cases by high-throughput sequencing (HTS), we detected a unique case of a clinical TBEV infection in a three-week old Dalmatian puppy from 2019 (Dawson et al. 2023). In the present case report, we followed up this finding and found that the other two littermates had similar neurological signs and neuropathological lesions and were also positive for TBEV by RT-qPCR. Our results suggest that the TBEV infection of the entire litter did not occur *via* tick-bites, but rather through a vector-independent transmission from the dam to the offspring.

## 2. Case presentation

In November 2019, a litter of three Dalmatian puppies was born after induced birth with aglepristone (progesterone receptor antagonist) in the Swiss canton of Thurgau. From birth on all three puppies showed weakness, trembling and an abnormally slow development. At two weeks of age, the first puppy (case ID ‘S19-1708’) was fed a commercial pasteurized goat’s milk product as an alternative nutrition source for rearing puppies, as it was no longer drinking milk from its mother. Neither the other two littermates nor the dam received the pasteurized goat’s milk, although contact cannot be fully excluded. The first puppy began to show epileptic seizures lasting ∼30 s four hours after being fed goat’s milk and was presented to a veterinary clinic the following day. Clinical presentation included multiple focal seizures around the eyes and ears, hypoglycaemia, and an elevated body temperature of 38.6 °C. Lymph nodes and cardiac examination were unremarkable. Due to the poor condition and on request of the owner, the puppy was euthanized, and the case was further investigated at the Institute of Veterinary Pathology of Zurich. Two days after the euthanasia of the first puppy, the other two littermates (‘S19-1722’, ‘S19-1723’) also developed more pronounced neurological signs including generalized seizures and were presented to the Small Animal Clinic of the Vetsuisse Faculty Zurich for further assessment. Examination of the cerebral spinal fluid (CSF) showed a mononuclear pleocytosis, suggesting an encephalitis. Due to uncontrollable seizures and further deterioration of the condition, the two puppies were euthanized and subjected to pathological examination. The patient history also noted that the dam developed a fever shortly after euthanasia of the entire litter, however no neurological signs were observed, and follow-up showed that the dam recovered completely.

**Figure 1.**
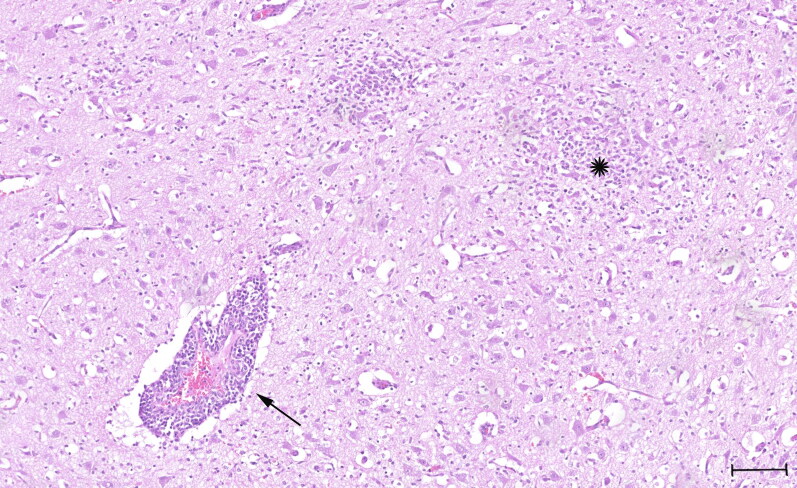
Histology slide stained with Hematoxylin and Eosin (H&E) showing prominent perivascular cuffing (arrow) with lymphocytes, few plasma cells and some macrophages and formation of multiple glial nodules (star). Quadrigeminal bodies, mesencephalon. Scale bar: 100 µm.

The findings at necropsy and of the histopathological examination of the three puppies were similar and included a severe lymphoplasmacellular and slightly purulent meningitis and leuko- and polioencephalitis with a mild lympho-histiocytic vasculitis, gliosis, glial nodule formation and beginning malacia (Figure 1). Furthermore, all three puppies were diagnosed with a moderate beginning interstitial pneumonia and two puppies (‘S19-1708’ and ‘S19-1723’) showed a mild purulent myocardial vasculitis. A moderate lymphocytic depletion of the lymph nodes was also noted in all three puppies. The findings were most consistent with a viral infection; however, subsequent PCR (canine herpesvirus 1) and immunohistochemistry for viral and bacterial infectious agents (parvovirus, *neospora caninum*, *toxoplasma gondii*, canine distemper virus, West Nile virus, *Leptospira interrogans* and *Listeria monocytogenes*) were negative.

In a retrospective metatranscriptomics study for detecting the viral cause of canine non-suppurative encephalitis of unknown viral origin, we applied high-throughput sequencing (HTS) to total RNA extracted from formalin-fixed and paraffin embedded (FFPE) material. We extracted total RNA from four 10 µm thick FFPE tissue samples using the RNeasy FFPE kit (Qiagen) after deparaffinization with xylol. FFPE blocks of the three puppies included brain tissue as well as FFPE blocks containing multiple peripheral organs, including the heart, lung, liver, kidney, intestinal tract, and lymphoid organs (primary and secondary). The extracted RNA from FFPE brain material of one puppy (‘S19-1723’) was submitted to the Next Generation Sequencing Platform of the Vetsuisse Faculty Bern for cDNA library preparation and HTS. The RNA concentration was assessed with the Qubit^TM^ 4 Fluorometer (Invitrogen) and quantified with the 5200 Fragment Analyzer System (Agilent). A cDNA library was prepared with the CORALL Total RNA-Seq Library kit (Lexogen) and sequenced in single-end mode for 100 cycles on an Illumina NovaSeq 6000 machine with a read depth of ∼100 Mio. The obtained HTS reads were analysed with an in-house established bioinformatics pipeline for the detection of viruses (Wüthrich et al. 2016): 99′864′689 raw reads were subjected to a quality control and trimming process using fastqc (Ver. 0.11.7) (Andrews 2010) and fastp (Ver. 0.12.5) (Chen et al. 2018), respectively. Quality selected and trimmed reads were mapped to the dog reference genome (Canis lupus familiaris 3.1) using STAR (Ver. 2.7.3a) (Dobin et al. 2013) and mapped reads were subsequently removed. The remaining 11’581’203 reads were mapped to the RefSeq viral genome database (accessed September 2022) for the detection of known viruses using bowtie2 (Ver. 2.3.4.1) (Langmead and Salzberg 2012). We identified 3’640 sequence reads mapping to the TBEV reference genome (GenBank accession NC_001672.1) with a nearly full-length genome coverage of 99.5% and a read depth of 23.4. In addition, we *de novo* assembled reads using SPAdes (Ver. 3.12.0) (Nurk et al. 2013) to generate contiguous sequences (contigs) and identified two TBEV contigs with a genome coverage of 97.5%. Using the Geneious Prime 2023.1 software package (https://www.geneious.com) we performed a reference guided assembly of mapped reads and contigs to generate a consensus sequence covering the full-length TBEV genome (GenBank Accession number OR523238). A BLASTn search (Ver. 2.10.1+) (Altschul et al. 1997) of the consensus sequence showed a best hit for the TBEV strain ‘Kuutsalo-14 Ixodes ricinus Finland-2017’ (GenBank Accession no. MG589938.1) (Kuivanen et al. 2018).

For the detection and confirmation of TBEV by RT-qPCR, we used the AgPath-ID One Step RT-qPCR System (Applied Biosystems^TM^) with a Taqman probe-based RT-qPCR assay and established primer-probe combinations targeting the envelope (E) gene (Gäumann et al. 2010). RT-qPCR was performed on RNA extracts from FFPE brain tissue of all three puppies as well as RNA extracts from FFPE blocks containing several peripheral organs (Table 1). Due to the retrospective nature of this study, further analysis of the CSF sample taken from one puppy for the presence of anti-TBEV antibodies was not possible.

**Table 1.** RT-qPCR results for tick-borne encephalitis virus (TBEV).

Animal-ID	Cq	Organ
S19-1708	21.54	Brain
	n.d.	Lung, liver, spleen, kidney
	n.d.	Heart, lung, bladder
	n.d.	Lung, intestine, thymus, pancreas, lymph node
S19-1722	19.70	Brain
	n.d.	Kidney, spleen, liver, lymph node
	37.84	Heart, lung, lymph node
	31.99	Intestine, thymus, lymph node, tonsil, bone marrow
S19-1723	23.75	Brain
	n.d.	Kidney, liver, lymph node
	29.17	Heart, lung
	33.16	Spleen, intestine, tonsil, thymus, lymph node

[n.d.] not detectable.

Shown are the cq values for RT-qPCR TBEV performed on total RNA extracted from the brain and extra-neural organs (FFPE tissues) of the three puppies. Extra-neural organs include RNA extracted from several tissue types, because they were embedded in the same FFPE tissue block.

**Figure 2.**
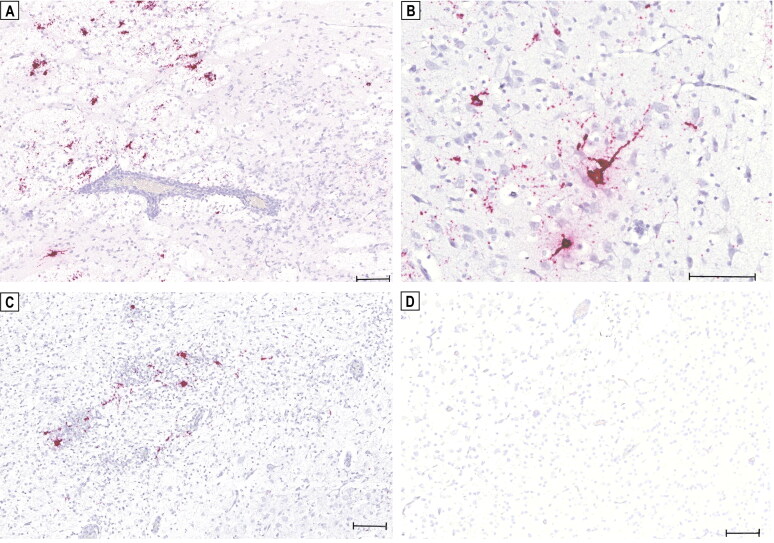
Detection of tick-borne encephalitis virus (TBEV) RNA in the brain of three Dalmatian puppies by *in situ* hybridization (ISH). Positive hybridization signals shown as red granular staining can be seen in the claustrum of puppy ‘S19-1708’ (a). Positive signals for TBEV RNA can be seen in the thalamus of puppy ‘S19-1722’ (B) and in the thalamus of puppy ‘S19-1723’ (C). No *in situ* detection of TBEV RNA in the brain of a healthy young dog serving as a negative control (D). Scale bars: 100 µm.

We demonstrated the presence of TBEV RNA by *in situ* hybridization (ISH) on FFPE tissue sections (3 µm) of brain and peripheral organs using an RNA ISH probe (V-TBEV-NS3; Advanced Cell Diagnostics; cat no. 575601) targeting the NS3 of the TBEV genome. ISH was performed with the RNAscope® 2.5 HD Reagent Kit-RED (Advanced Cell Diagnostics). TBEV RNA could be detected by ISH in the soma of neurons and glial cells as well as along axonal structures in all three brain samples, while the abundance of positive ISH signals varied in all three puppies (Figure 2). However, in contrast to the RT-qPCR results, no clear positive signals for TBEV RNA were detected in the peripheral organs.

**Figure 3.**
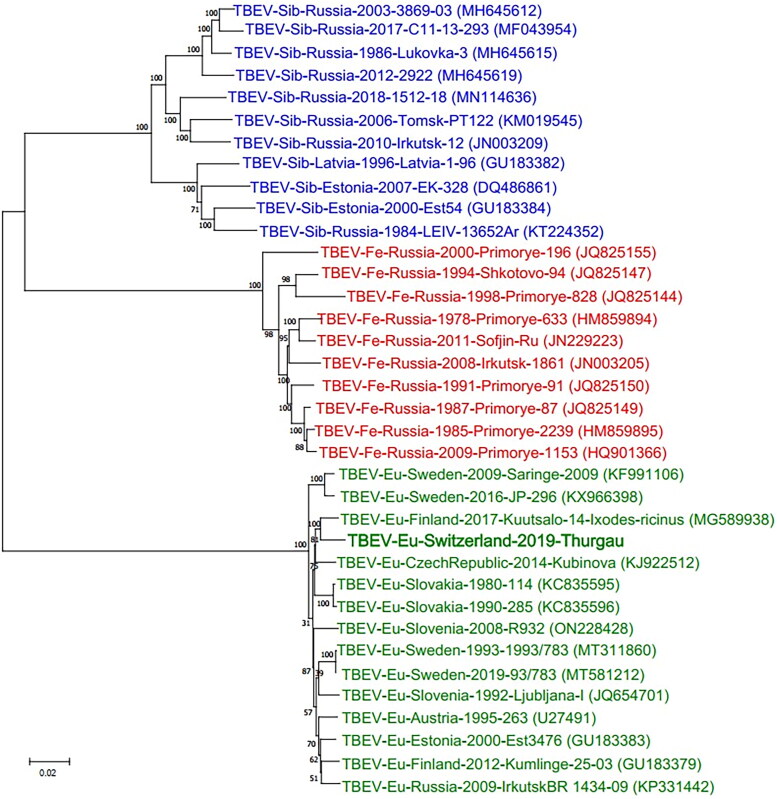
Phylogenetic comparison of whole genome sequences of representative tick-borne encephalitis virus (TBEV) strains from the three main subtypes. Representative full-length sequences were obtained from NCBI GenBank with corresponding accession numbers shown in brackets. Colors indicate the three different TBEV subtypes (green: European subtype [TBEV-Eu], red: Far Eastern subtype [TBEV-Fe], blue: Siberian subtype [TBEV-Sib]). The obtained consensus sequence of puppy ‘S19-1723’, TBEV-Eu-Switzerland-2019-Thurgau (bold), clusters with the European subtypes.

Lastly, we conducted a phylogenetic analysis for the obtained TBEV consensus sequence (‘S19-1723’) based on the complete genome in comparison to those of various representative TBEV reference strains obtained from NCBI GenBank from the three main TBEV subtypes: European (TBEV-Eu), Far Eastern (TBEV-Fe) and Siberian (TBEV-Sib). All sequences were aligned using the MAFFT (Ver. 7.475) (Katoh and Standley 2013) software. A maximum-likelihood tree was constructed with IQ-Tree (Ver. 2.0.3) (Minh et al. 2020) with 1’000 bootstrap replicates. The phylogenetic tree showed a clustering of our TBEV strain TBEV-Eu-Switzerland-2019-Thurgau with other strains of the TBEV-Eu subtype (Figure 3). Using the same approach, we also conducted a phylogenetic analysis based on the complete sequence of the E gene with TBEV strains isolated from ticks from various Swiss cantons and found a clustering of our strain with Swiss isolates originating from the same regions (Supplemental Figure S1).

## 3. Discussion

The main transmission route of TBEV is vector dependent *via* ticks of the genus *Ixodes* (Chitimia-Dobler et al. 2021). Given the young age of the puppies of only two and three weeks, it seems rather unlikely that a transmission occurred *via* ticks. Therefore, we propose that the three puppies were infected with TBEV through an alternative vector-independent route. This assumption is further supported by the lack of tick exposure mentioned in the patients’ clinical histories and also the time of year outside the seasonal activity of ticks in Switzerland; although a transmission *via* ticks cannot be fully excluded, especially in the TBEV endemic Swiss canton of Thurgau (Krech 2002; Chitimia-Dobler et al. 2021; Desgrandchamps and Posfay-Barbe 2021; Federal Office of Public Health (FOPH) 2023). Alimentary transmission of TBEV through the consumption of milk from infected animals can also lead to severe illness in dogs, although less frequent (Klimes et al. 2001; Ličková et al. 2021). A prepared goat’s milk supplement for rearing puppies was fed to one puppy (‘S19-1708’), which later began to show cramping and spasming. Alimentary TBEV infections in dogs can occur through the intake of raw infected goat’s milk (Klimes et al. 2001). However, it is rather unlikely to be the source of infection, since the commercially available goat’s milk was subjected to a standardized pasteurisation process and was only fed to one of the affected puppies.

Due to climate changes with increasing temperatures and milder winters, tick infestations and tick-borne illnesses in animals and humans have shifted in central Europe from a seasonal towards an all-year occurrence (Andersson et al. 2020; Probst et al. 2023; Federal Office of Public Health (FOPH) 2023). According to Swiss weather reports, the month of October in the year 2019 was the fifth warmest October since the beginning of systematic temperature recordings in 1864 (Meteo Switzerland 2019). The unusually mild temperatures measured in the second half of October, reaching up to 25 °C during the day, likely resulted in enhanced activity in ticks. A few weeks later, the dam developed a fever shortly after the disease of the puppies. Fever is an unspecific but common sign observed in dogs infected with TBEV (Pfeffer et al. 2021). Although no ticks were observed on the dam, the lack of prophylactic measures taken against ticks in an area endemic to TBEV would suggest that an asymptomatic TBEV infection through a tick bite took place and that the virus was transmitted from the dam to the puppies during the critical short viremic phase. A serological test result of the dam was communicated to us by the owner during the preparation of this manuscript. The test was conducted 4 years after the disease occurred in the offspring and confirmed the presence of TBEV specific IgGs (conducted at IDEXX Diavet, 8806 Baech SZ, Switzerland) by ELISA with 380 Vienna units (VIEU)/ml (cut off > 126 VIEU/ml). Together with the medical history, there is good evidence that there was a transmission of the virus from the dam to the puppies. Since dogs are accidental hosts for TBEV and do not transmit the virus directly to other animals, a possible transmission of TBEV from the dam to the puppies would likely occur intrauterine, or post-partum during lactation (Chitimia-Dobler et al. 2021; Pfeffer et al. 2021). Transmission of TBEV during lactation has been described for humans with a recent case report of an unvaccinated mother infected with TBEV transmitting the virus to her newborn *via* breast milk (Kerlik et al. 2022). Similar observations have also been made in small rodents as naturally TBEV infected reservoir hosts (Bakhvalova et al. 2009). However, the abnormal neurological status of the puppies from birth on suggests that a materno-fetal transmission already occurred intrauterine, which led to a severe and fulminant TBEV infections in the three puppies. In addition, congenital cerebrospinal hypomyelination, which can be a cause for the trembling symptoms of the Dalmatian puppies, could be ruled out based on histopathological examinations (Greene et al. 1977).

Several arboviruses of the genus *Flavivirus*, including Zika virus, harbour teratogenic properties and can lead to severe and fatal neonatal infections after vertical virus transmission from infected pregnant women (Charlier et al. 2017). There is currently no evidence available suggesting a materno-fetal transmission of TBEV occurring in humans and there are no such reports of vertical transmission in dogs (Charlier et al. 2017; Divé et al. 2020; Pfeffer et al. 2021). The family *Flaviviridae* includes other important viruses where vertical transmission routes play a significant role, e.g. bovine viral diarrhoea virus (BVDV) of the genus *Pestivirus*. Overall, the time point of the infection and subsequent vertical virus transfer are crucial for the outcome of the disease in the offspring.

Moreover, RT-qPCR performed on total RNA extracts from FFPE blocks containing multiple peripheral organs showed the presence of the virus outside of the CNS in two of the three littermates. However, our ISH approach was not able to detect a clear positive signal for TBEV RNA in the peripheral organs, which could be due to the low viral concentration or the rapid clearance of the virus (Weissenböck et al. 1998; Pfeffer et al. 2021). In contrast to humans, TBEV infections in dogs are not associated with histopathological lesions in visceral organs (Salát and Růžek 2020; Borde and Zajkowska 2021). The detection of TBEV in peripheral organs of the puppies may reflect the severity of the clinical course that might have been accentuated as a result of an inadequate immune response from the immature immune system of the three puppies.

## 4. Conclusions

Overall, our findings shed light on alternative vector-independent transmission routes of TBEV infections in dogs. We highlight the importance of prophylactic measures for dogs against ticks based on the guidelines provided by the European Scientific Counsel Companion Animal Parasites (ESCCAP, www.esccap.ch) and we recommend veterinary practitioners to consider TBEV as a differential diagnosis in dogs suffering from neurological signs.

## Supplementary Material

Supplemental Material

## Data Availability

Raw sequencing reads generated and analysed are available at the National Center for Biotechnical Information (NCBI) sequence read archive under the Accession Number PRJNA1014446. The genome of TBEV-EU-Switzerland-2019-Thurgau is available at NCBI GenBank under the Accession Number OR523238.

## References

[CIT0001] Altschul SF, Madden TL, Schäffer AA, Zhang J, Zhang Z, Miller W, Lipman DJ. 1997. Gapped BLAST and PSI-BLAST: a new generation of protein database search programs. Nucleic Acids Res. 25(17):3389–3402. doi: 10.1093/nar/25.17.3389. 9254694 PMC146917

[CIT0002] Andersson E, Kendall A, Url A, Auer A, Leschnik M. 2020. The first RT-qPCR confirmed case of tick-borne encephalitis in a dog in Scandinavia. Acta Vet Scand. 62(1):51. doi: 10.1186/s13028-020-00550-2. 32912238 PMC7488111

[CIT0003] Andrews S. 2010. FastQC: a quality control tool for high throughput sequence data. http://www.bioinformatics.babraham.ac.uk/projects/fastqc/ (accessed August 2023).

[CIT0004] Bakhvalova VN, Potapova OF, Panov VV, Morozova OV. 2009. Vertical transmission of tick-borne encephalitis virus between generations of adapted reservoir small rodents. Virus Res. 140(1-2):172–178. doi: 10.1016/j.virusres.2008.12.001. 19111585

[CIT0005] Borde JP, Zajkowska J. 2021. TBE in adults. Chapter 5. In: Dobler G, Erber W, Bröker M, Schmitt HJ, eds. The TBE book. 4th ed. Singapore: Global Health Press.

[CIT0006] Charlier C, Beaudoin MC, Couderc T, Lortholary O, Lecuit M. 2017. Arboviruses and pregnancy: maternal, fetal, and neonatal effects. Lancet Child Adolesc Health. 1(2):134–146. doi: 10.1016/S2352-4642(17)30021-4. 30169203

[CIT0007] Chen S, Zhou Y, Chen Y, Gu J. 2018. fastp: an ultra-fast all-in-one FASTQ preprocessor. Bioinformatics. 34(17):i884–i890. doi: 10.1093/bioinformatics/bty560. 30423086 PMC6129281

[CIT0008] Chitimia-Dobler L, Mackenstedt U, Kahl O. 2021. Transmission/natural cycle. Chapter 3. In: Dobler G, Erber W, Bröker M, Schmitt HJ, eds. The TBE book. 4th ed. Singapore: Global Health Press. p. 47–63.

[CIT0009] Dawson KLD, Wildi N, Koch MC, Oevermann A, Rosato G, Grest P, Hilbe M, Seuberlich T. 2023. Virus discovery in dogs with non-suppurative encephalitis reveals a high incidence of tick-borne encephalitis virus infections in Switzerland. Schweiz Arch Tierheilkd. 165(10):656–666. 37822248 10.17236/sat00407

[CIT0010] Desgrandchamps D, Posfay-Barbe MK. 2021. TBE in Switzerland and Liechtenstein. Chapter 12b. In: Dobler G, Erber W, Bröker M, Schmitt HJ, eds. The TBE Book. 4th ed. Singapore: Global Health Press. p. 342–347.

[CIT0011] Deviatkin AA, Kholodilov IS, Vakulenko YA, Karganova GG, Lukashev AN. 2020. Tick-borne encephalitis virus: an emerging ancient zoonosis? Viruses. 12(2):247. doi: 10.3390/v12020247. 32102228 PMC7077300

[CIT0012] Divé I, Veje M, Dobler G, Bergström T, Buxmann H, Paul B, Louwen F, Berger A, Jahnke K, Strzelczyk A, et al. 2020. Tick-borne encephalitis virus (TBEV) infection in pregnancy: absence of virus transmission to the fetuses despite severe maternal disease – A case study. Ticks Tick Borne Dis. 11(5):101491. doi: 10.1016/j.ttbdis.2020.101491. 32723645

[CIT0013] Dobin A, Davis CA, Schlesinger F, Drenkow J, Zaleski C, Jha S, Batut P, Chaisson M, Gingeras TR. 2013. STAR: ultrafast universal RNA-seq aligner. Bioinformatics. 29(1):15–21. doi: 10.1093/bioinformatics/bts635. 23104886 PMC3530905

[CIT0014] Federal Office of Public Health (FOPH). 2023. Frühsommer-Meningoenzephalitis (FSME). https://www.bag.admin.ch/bag/de/home/krankheiten/krankheiten-im-ueberblick/fsme.html.

[CIT0015] Gäumann R, Mühlemann K, Strasser M, Beuret CM. 2010. High-throughput procedure for tick surveys of tick-borne encephalitis virus and its application in a national surveillance study in Switzerland. Appl Environ Microbiol. 76(13):4241–4249. doi: 10.1128/AEM.00391-10. 20453126 PMC2897458

[CIT0016] Greene CE, Vandevelde M, Hoff EJ. 1977. Congenital cerebrospinal hypomyelinogenesis in a pup. J Am Vet Med Assoc. 171(6):534–536. 562333

[CIT0017] Katoh K, Standley DM. 2013. MAFFT multiple sequence alignment software version 7: improvements in performance and usability. Mol Biol Evol. 30(4):772–780. doi: 10.1093/molbev/mst010. 23329690 PMC3603318

[CIT0018] Kerlik J, Avdičová M, Musilová M, Bérešová J, Mezencev R. 2022. Breast milk as route of tick-borne encephalitis virus transmission from mother to infant. Emerg Infect Dis. 28(5):1060–1061. doi: 10.3201/eid2805.212457. 35447060 PMC9045420

[CIT0019] Klimes J, Juricová Z, Literák I, Schánilec P, Trachta e Silva E. 2001. Prevalence of antibodies to tickborne encephalitis and West Nile flaviviruses and the clinical signs of tickborne encephalitis in dogs in the Czech Republic. Vet Rec. 148(1):17–20. doi: 10.1136/vr.148.1.17. 11200400

[CIT0020] Krech T. 2002. TBE foci in Switzerland. Int J Med Microbiol. 291 Suppl 33:30–33. doi: 10.1016/s1438-4221(02)80006-6. 12141754

[CIT0021] Kuivanen S, Smura T, Rantanen K, Kämppi L, Kantonen J, Kero M, Jääskeläinen A, Jääskeläinen AJ, Sane J, Myllykangas L, et al. 2018. Fatal tick-borne encephalitis virus infections caused by Siberian and European subtypes, Finland, 2015. Emerg Infect Dis. 24(5):946–948. doi: 10.3201/eid2405.171986. 29664395 PMC5938788

[CIT0022] Kunze M, Erber W, Haditsch M. 2021. TBE as a matter of public health. Chapter 13. In: Dobler G, Erber W, Bröker M, Schmitt HJ, eds. The TBE book. 4th ed. Global Health Press, Singapore. p. 359–366.

[CIT0023] Langmead B, Salzberg SL. 2012. Fast gapped-read alignment with Bowtie 2. Nat Methods. 9(4):357–359. doi: 10.1038/nmeth.1923. 22388286 PMC3322381

[CIT0024] Leschnik MW, Kirtz GC, Thalhammer JG. 2002. Tick-borne encephalitis (TBE) in dogs. Int J Med Microbiol. 291 Suppl 33:66–69. doi: 10.1016/s1438-4221(02)80014-5. 12141763

[CIT0025] Ličková M, Fumačová Havlíková S, Sláviková M, Klempa B. 2021. Alimentary Infections by tick borne encephalitis virus. Viruses. 14(1):56. doi: 10.3390/v14010056. 35062261 PMC8779402

[CIT0026] Meteo Switzerland. 2019. Klimabulletin Oktober 2019. Zürich. (German).

[CIT0027] Minh BQ, Schmidt HA, Chernomor O, Schrempf D, Woodhams MD, von Haeseler A, Lanfear R. 2020. IQ-TREE 2: new models and efficient methods for phylogenetic inference in the genomic era. Mol Biol Evol. 37(5):1530–1534. doi: 10.1093/molbev/msaa015. 32011700 PMC7182206

[CIT0028] Nurk S, Bankevich A, Antipov D, Gurevich AA, Korobeynikov A, Lapidus A, Prjibelski AD, Pyshkin A, Sirotkin A, Sirotkin Y, et al. 2013. Assembling single-cell genomes and mini-metagenomes from chimeric MDA products. J Comput Biol. 20(10):714–737. doi: 10.1089/cmb.2013.0084. 24093227 PMC3791033

[CIT0029] Pfeffer M, Dobler G. 2011. Tick-borne encephalitis virus in dogs–is this an issue? Parasit Vectors. 4:59. 21489255 10.1186/1756-3305-4-59 PMC3094398

[CIT0030] Pfeffer M, Schmuck HM, Leschnik M. 2021. Chapter 8: TBE in animals. In: Dobler G, Erber W, Bröker M, Schmitt HJ, eds. The TBE book. 4th ed. Singapore: Global Health Press. p. 105–118.

[CIT0031] Probst J, Springer A, Topp AK, Bröker M, Williams H, Dautel H, Kahl O, Strube C. 2023. Winter activity of questing ticks (Ixodes ricinus and Dermacentor reticulatus) in Germany – Evidence from quasi-natural tick plots, field studies and a tick submission study. Ticks Tick Borne Dis. 14(6):102225. doi: 10.1016/j.ttbdis.2023.102225. 37399628

[CIT0032] Ruzek D, Avšič Županc T, Borde J, Chrdle A, Eyer L, Karganova G, Kholodilov I, Knap N, Kozlovskaya L, Matveev A, et al. 2019. Tick-borne encephalitis in Europe and Russia: review of pathogenesis, clinical features, therapy, and vaccines. Antiviral Res. 164:23–51. doi: 10.1016/j.antiviral.2019.01.014. 30710567

[CIT0033] Salat J, Hunady M, Schanilec P, Strakova P, Stefanik M, Svoboda P, Strelcova L, Bojcukova J, Palus M, Růžek D. 2021. Experimental and natural infections of tick-borne encephalitis virus in dogs. Viruses. 13(10):2039. doi: 10.3390/v13102039. 34696468 PMC8537875

[CIT0034] Salát J, Růžek D. 2020. Tick-borne encephalitis in domestic animals. Acta Virol. 64(2):226–232. 32551790 10.4149/av_2020_212

[CIT0035] Schuler M, Zimmermann H, Altpeter E, Heininger U. 2014. Epidemiology of tick-borne encephalitis in Switzerland, 2005 to 2011. Euro Surveill. 19(13):20756. 24721541 10.2807/1560-7917.es2014.19.13.20756

[CIT0036] Simmonds P, Becher P, Bukh J, Gould EA, Meyers G, Monath T, Muerhoff S, Pletnev A, Rico-Hesse R, Smith DB, Stapleton JT. 2017. ICTV virus taxonomy profile: flaviviridae. J Gen Virol. 98(1):2–3. doi: 10.1099/jgv.0.000672. 28218572 PMC5370391

[CIT0037] Weissenböck H, Suchy A, Holzmann H. 1998. Tick-borne encephalitis in dogs: neuropathological findings and distribution of antigen. Acta Neuropathol. 95(4):361–366. doi: 10.1007/s004010050811. 9560013

[CIT0038] Wüthrich D, Boujon CL, Truchet L, Selimovic-Hamza S, Oevermann A, Bouzalas IG, Bruggmann R, Seuberlich T. 2016. Exploring the virome of cattle with non-suppurative encephalitis of unknown etiology by metagenomics. Virology. 493:22–30. doi: 10.1016/j.virol.2016.03.009. 26994586

