# Supplementary material for: Fatal tick-borne encephalitis virus infection in Dalmatian puppy-dogs after putative vector independent transmission
Source: Vet Q. 2024 Apr 10;44(1):1–7. doi: 10.1080/01652176.2024.2338385 (PMC11008312; doi:10.1080/01652176.2024.2338385)
Supplement: Supplemental Material [file TVEQ_A_2338385_SM5173.zip › Supplementary Figure caption/Supplementary Figure caption.docx]

**Supplementary Figure S1:** Phylogenetic comparison of the complete nucleotide (nt) sequence of the envelope (E) gene of Swiss tick-borne encephalitis virus (TBEV) isolates. The full nt sequence of the E gene from representative Swiss TBEV isolates were obtained from NCBI GenBank with corresponding accession numbers shown in brackets. Our obtained TBEV strain shows a close relation to TBEV isolates from nearby geographical region.
