# Supplementary figures and images for: Fatal tick-borne encephalitis virus infection in Dalmatian puppy-dogs after putative vector independent transmission
Source: Vet Q. 2024 Apr 10;44(1):1–7. doi: 10.1080/01652176.2024.2338385 (PMC11008312; doi:10.1080/01652176.2024.2338385)

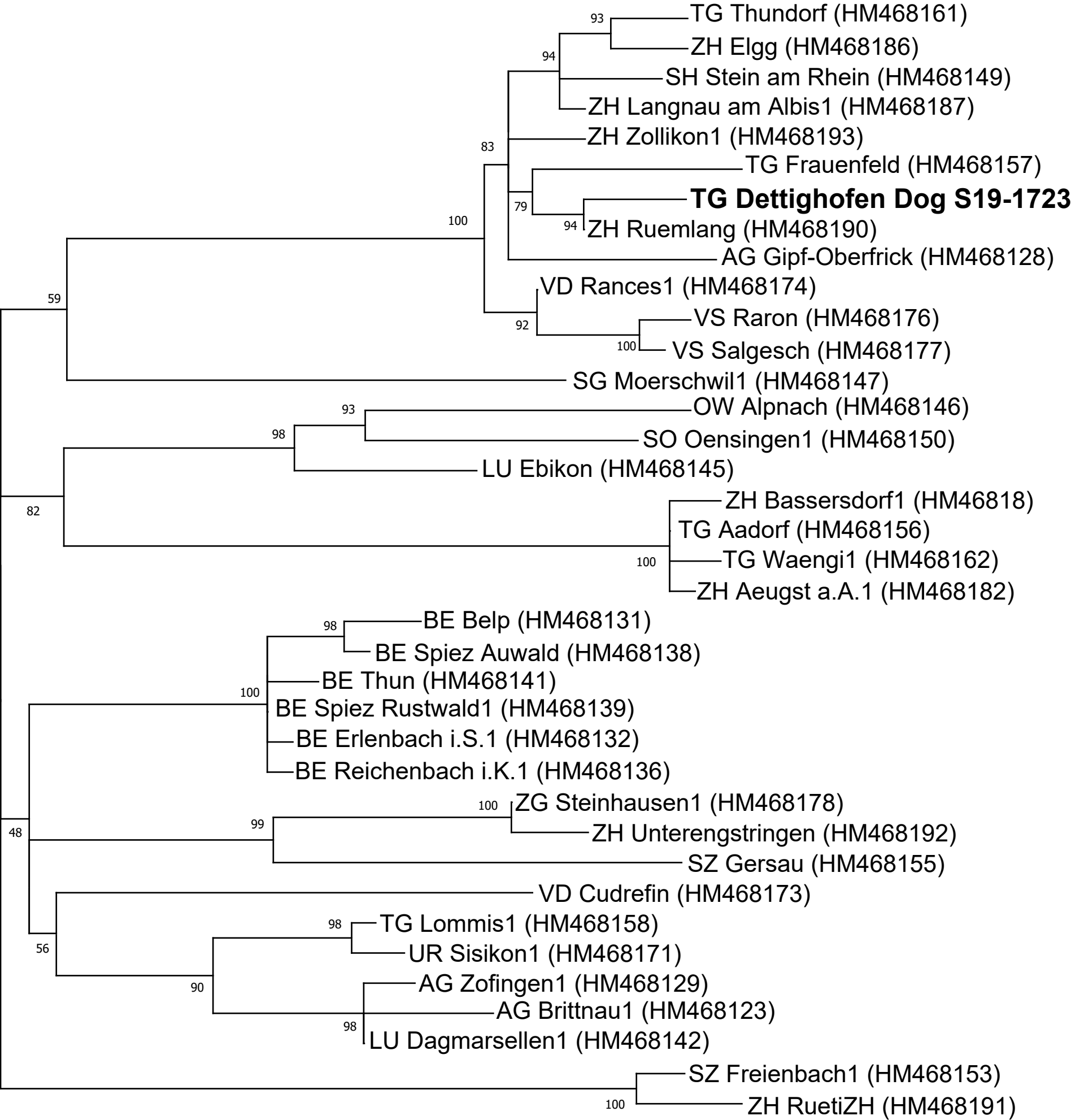

Supplement: Supplemental Material [file TVEQ_A_2338385_SM5173.zip › Supplementary Figure caption/Figure_S1_NEW.pdf]
